# Unravelling the role of TET2 in venous thromboembolism: insights into mechanisms and therapeutic implications

**DOI:** 10.3389/fcvm.2025.1577303

**Published:** 2025-10-15

**Authors:** Yujie Bai, Xiao Wang, Ning Lin, Rongyuan Li, Weilin Hu, Jiayu Wang, Chun Chen, Jie Liu, Jian Feng, Fuxiang Li

**Affiliations:** ^1^ Department of Respiratory Medicine, School of Clinical Medicine, Southwest Medical University, Luzhou, Sichuan, China; ^2^ Department of Critical Care Medicine, The General Hospital of Western Theater Command, Chengdu, Sichuan, China; ^3^ Department of Hematology, The General Hospital of Western Theater Command, Chengdu, Sichuan, China; ^4^ Department of Nutrition, The General Hospital of Western Theater Command, Chengdu, Sichuan, China; ^5^ Department of Pain Medicine, The General Hospital of Western Theater Command, Chengdu, Sichuan, China; ^6^ College of Medicine, Southwest Jiaotong University, Chengdu, Sichuan, China

**Keywords:** TET2, venous thromboembolism, molecular mechanisms, demethylation, therapeutic strategies

## Abstract

The development of venous thromboembolism (VTE) may complicate not only the management of the primary disease but also significantly affect the overall quality of life and prognosis for the patient. With the increasing understanding of the incidence and risks associated with VTE, there is an imminent need for better management strategies, coupled with a wider knowledge base. In recent years, Ten-Eleven Transformation 2 (TET2) has become a subject of interest among medical scientists because of its function as DNA demethylase for the treatment of a number of hematologic and oncologic disorders. The current literature concerning the association of VTE with mutations in the TET2 gene is rather diverse in terms of outcomes and, therefore, not completely coherent. While some papers propose that TET2 has an antithrombotic effect, others point to a prothrombotic effect or a more subtle effect of TET2 on the development of thromboembolism. These different views must then be integrated in order to create the aetiologic narrative of TET2 in VTE that provides a framework for understanding the epidemiologic and clinical realities. However, there is no review on the mechanism and clinical significance of TET2 in venous thromboembolism. In this review article, the authors strived to investigate Ten-Eleven Transformation 2 (TET2) connected with venous thromboembolism (VTE), analyze its molecular mechanism features and draw clinical conclusions. It is the purpose of this work to perform a comprehensive review of the TET2 function, to elucidate its involvement in VTE development, and to discuss possible treatments based on targeting TET2. It is our understanding that the review of the current literature will offer fresh insight and research agendas for the future endeavours and practice of the significant medical speciality.

## 1 Introduction

Venous thromboembolism (VTE) is a major clinical challenge, particularly among hospitalized patients with malignancy as comorbidity. VTE encompasses deep vein thrombosis (DVT) and pulmonary embolism (PE), both serious health sequelae and potentially life-threatening conditions. VTE occurs across an incredibly broad patient population, but the incidence is strikingly elevated in cancer patients because of factors such as tumour burden, surgical procedures, chemotherapy, and many others that greatly elevate the risk of VTE (1). However, the development of VTE may complicate not only the management of the primary disease but also significantly affect the overall quality of life and prognosis for the patient (2). With the increasing understanding of the incidence and risks associated with VTE, there is an imminent need for better management strategies, coupled with a wider knowledge base. The TET2 gene, a member of the Ten-Eleven Translocation (TET) family, is highly associated with controlling the DNA methylation process and has been identified as an important factor in a range of haematological malignancies. TET2 catalyzes the conversion of 5-methylcytosine into 5-hydroxymethylcytosine, which is an important epigenetic modification that influences gene expression and cellular differentiation (3). Indeed, mutations of the TET2 gene frequently occur in myeloid malignancies, including acute myeloid leukemia (AML) and chronic myeloid leukemia (CMML), among others, and are associated with a worse prognosis (4). However, the exact function of TET2 in hematopoietic processes and, therefore, possible involvement in venous thromboembolism (VTE), especially in malignancies, remains an active area of research. Its determination is of critical importance because of the complex interrelation between genetic risk factors such as those involving the mutation of the TET2 gene and pathophysiological mechanisms of VTE. Such a detailing of the mechanisms of TET2 dysregulation leading to thrombotic events during the course of the research may unravel new avenues for therapy and better risk stratification of patients prone to VTE. This review outlines the relationship between TET2 and VTE and deepens the understanding of this important link in biology.

### 2.1 Biological functions of TET2

#### 2.1.1 Structure and mechanism of TET2

**Figure 1:**
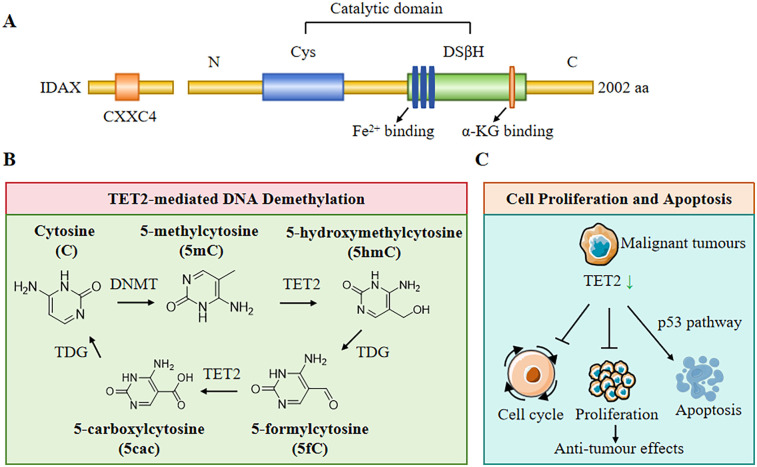
Structure and biological functions of TET2. **(A)** The structure of TET2 protein includes CXXC4 domain, cysteine (Cys)-rich domain, and a double-stranded β-helix fold (DSβH), which is characteristic of three Fe^2+^ binding and one α-Ketoglutarate (α-KG) binding. **(B)** TET2 plays a pivotal role in DNA demethylation. DNA methyltransferases (DNMT) convert cytosine **(C)** to form 5-methylcytosine (5mC), then TET2 and thymine DNA glycosylase (TDG) convert 5mC to 5-hydroxymethylcytosine (5hmC), 5-formylcytosine (5fC), and 5-carboxylcytosine (5cac). **(C)** TET2 plays a central role in regulating cell proliferation, cell cycle, and cell apoptosis.

The TET2 gene is a tumour suppressor located on chromosome 4q24 and is a member of the TET enzyme family (5). The TET2 gene consists of 11 exons and plays an essential role in the regulation of DNA methylation and hydroxymethylation. Structurally, TET2 contains a conserved C-terminal structural domain, which is also its functional domain. Moreover, the cysteine-rich domain and the double-stranded *β*-helix folding domain confer its dioxygenase activity, which is essential for catalyzing the conversion of 5-methylcytosine to 5-hydroxymethylcytosine (5-hmC) and further to 5-formylcytosine and 5-carboxylcytosine. This process is vital for active DNA demethylation, a mechanism that influences gene expression and cellular identity during development and in response to environmental cues (6). TET2 is a Fe^2+^- and *α*- ketoglutarate dependent (*α*KG dependent) DNA dioxygenase that mediates CpG demethylation of promoters and enhancers in hematopoietic progenitor cells and stem cells (HSPCs) (Figure 1A). Therefore, TET controls HSPC amplification and differentiation by altering gene expression patterns (7). The enzymatic activity of TET2 is regulated by various post-translational modifications, such as phosphorylation and ubiquitination, which modulate its stability and function (8). Importantly, TET2's role is not limited to oxidizing 5-methylcytosine (5mC) to 5-hydroxymethylcytosine (5hmC) and promoting DNA demethylation at the DNA level; it is also involved in the recruitment of transcriptional co-factors and chromatin remodelers, thereby influencing chromatin architecture and gene expression patterns (9). At the post-transcriptional level, TET2 can also catalyze RNA 5hmC modification, leading to instability and eventual degradation of the target RNA (10). Further research should be conducted on the structural and mechanistic basis of TET2 function to elucidate its broader implications in health and disease, particularly in haematological malignancies where TET2 mutations are prevalent (11).

#### 2.1.2 Role of TET2 in DNA demethylation

TET2 plays a pivotal role in the active demethylation of DNA, which is involved in cellular reprogramming and differentiation. The enzyme catalyzes the conversion of 5-methylcytosine to 5-hydroxymethylcytosine, which can subsequently be oxidized to 5-formylcytosine and 5-carboxylcytosine, leading to the removal of methyl groups through base excision repair mechanisms (Figure 1B) (12). This activity is particularly important during embryonic development and in the maintenance of pluripotency in stem cells, requiring precise regulation of DNA methylation patterns (13). Moreover, TET2-mediated demethylation has been shown to influence gene expression in various contexts, including the regulation of oncogenes and tumour suppressor genes in cancer (14). Additionally, TET2's involvement in the modulation of DNA methylation patterns has implications for metabolic processes, as seen in studies linking TET2 activity to glucose metabolism and insulin signaling (15). Therefore, TET2 assumes multiple functions in DNA demethylation, impacting not only gene regulation but also broader physiological processes.

#### 2.1.3 Relationship between TET2 and cell proliferation and apoptosis

The TET2 gene often undergoes mutations in malignant tumours and solid cancers of the hematopoietic system and is one of the most common mutated genes in clonal hematopoiesis in the general population (16). The influence of TET2 on cell proliferation and apoptosis is a critical aspect of its biological function, particularly in the context of cancer (Figure 1C). A previous study revealed that TET2 exerts tumour-suppressive effects by regulating cell cycle progression and promoting apoptosis in various cancer types, including leukaemia and solid tumours (14). For instance, restoration of TET2 function in TET2-deficient cancer cells is associated with reduced tumour growth and enhanced sensitivity to chemotherapeutic agents (17). Mechanistically, TET2 modulates the expression of genes involved in apoptosis and cell cycle regulation, such as those related to the p53 pathway and other apoptotic signalling cascades (18). Additionally, TET2 plays a central role in maintaining genomic stability and preventing clonal expansion of hematopoietic stem cells, which further underscores its importance in regulating cell proliferation and apoptosis (19). The interplay between TET2, cell proliferation, and apoptosis highlights its potential as a therapeutic target in cancer treatment, where modulation of TET2 activity could enhance the efficacy of existing therapies and improve patient outcomes.

### 2.2 The role of TET2 in venous thrombosis formation

#### 2.2.1 The association between TET2 and platelet function

**Figure 2:**
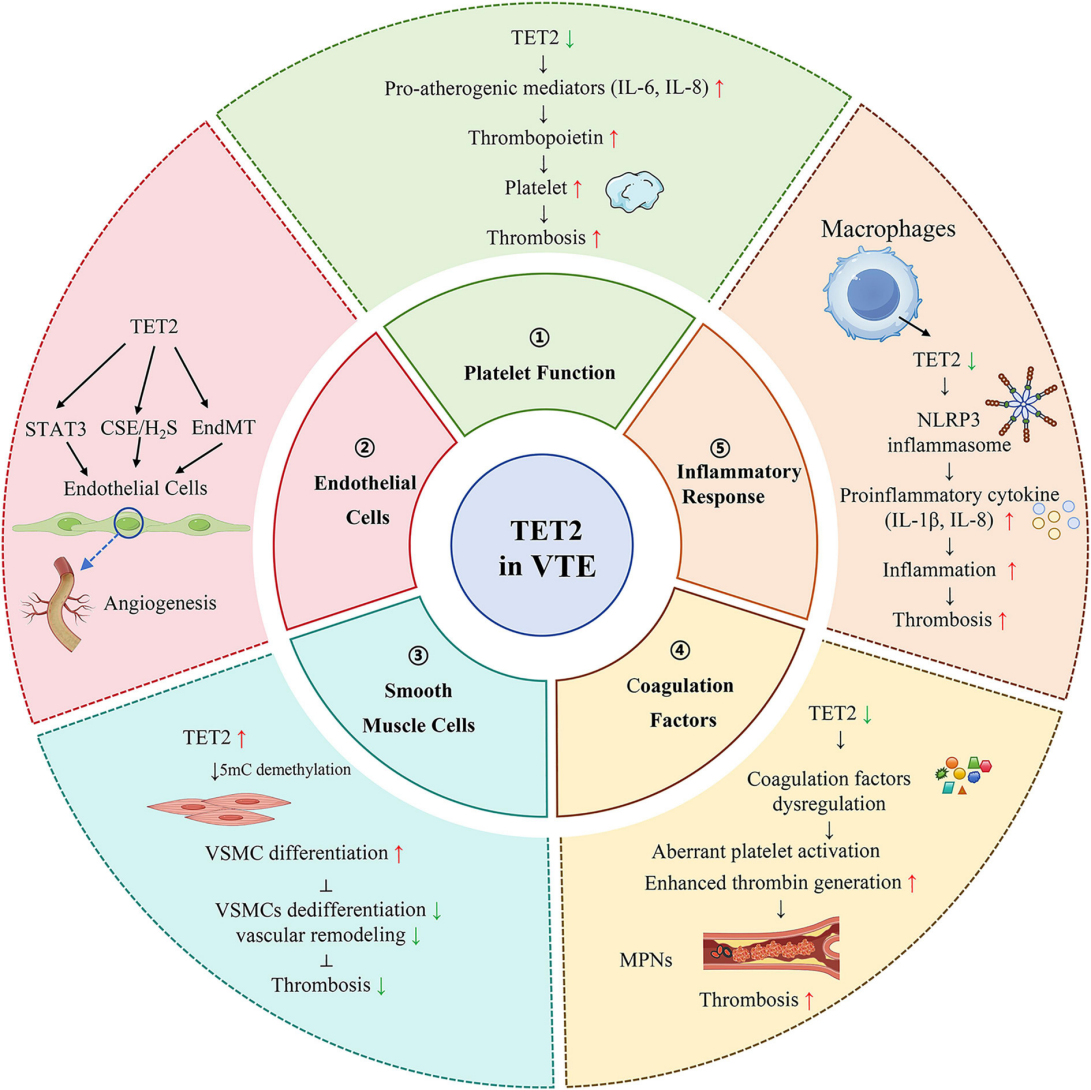
The role of TET2 in venous thromboembolism (VTE). TET2 plays an essential role in regulating platelet function (①), endothelial cell function (②), vascular smooth muscle cells (VSMCs) differentiation (③), coagulation factor expression (④), and inflammatory response (⑤).

TET2 (Ten-Eleven Translocation 2) plays an essential role in regulating platelet function, which is involved in venous thrombosis (Figure 2). Research has shown that the absence of TET2 can lead to decreased platelet function, which in turn impairs platelet activation and aggregation (10). These findings highlight its importance in maintaining normal hemostatic function. Specifically, TET2 deficiency in mice resulted in altered platelet responses to agonists, suggesting that TET2 is essential for optimal platelet function. Hence, TET2 deficiency may contribute to thrombotic risk. Furthermore, TET2 is involved in the epigenetic regulation of genes associated with platelet activation, underscoring its role in thrombogenesis. Aref et al. observed that platelet counts in TET2 mutant patients were higher than those in non-mutant patients (20).In contrast, Panuzzo et al. (2020) and Wang et al. (2019) observed that platelet counts in TET2 mutant patients were lower than those in non-mutant patients (21, 22). Veninga et al. (2020) explained higher platelet count in TET2 mutated patients as the TET gene product represses the transcription of inflammatory molecules, such as interleukin-6 and -8, which are known as pro-atherogenic mediators (23). So, somatic loss-of-function mutations in TET2 are associated with an increased inflammation tendency that subsequently up-regulates the thrombopoietin production by the liver resulting in higher platelet count. This regulatory function suggests that TET2 mutations or dysregulation could predispose individuals to thrombotic events, particularly in conditions such as myeloproliferative neoplasms, where thrombosis risk is notably elevated (24). Clinical data shows that CMML patients with TET2 mutations have lower platelet levels compared to wild-type TET2 patients (25, 26). In addition, previous studies using a genetically engineered TET2 deletion mouse model have found that the absence of TET2 results in a decreased proportion of megakaryocyte-erythroid progenitor cells and hyperploid megakaryocytes (10). This may be the mechanism by which TET2 affects platelet numbers and function.

#### 2.2.2 The role of TET2 in endothelial cells

TET2 also plays a pivotal role in endothelial cell function, influencing angiogenesis and vascular homeostasis (Figure 2). Research indicates that loss of TET2 impairs endothelial cell angiogenesis by downregulating key target genes associated with the STAT3 signalling pathway, which participates in endothelial cell proliferation and survival (27). Another study revealed that TET2 overexpression increases CSE expression by promoting demethylation of the CSE (cystathionine gamma-lyase) promoter, thereby upregulating the CSE/H2S system to protect endothelial function (28). Additionally, TET2 has been implicated in the endothelial mesenchymal transition (EndMT), a process that can contribute to vascular remodelling and thrombosis. Moreover, low shear stress conditions have been shown to downregulate TET2, thereby promoting endothelial-mesenchymal transition (EndMT) or inducing pyroptosis in endothelial cells, which may further exacerbate the risk of thrombosis (29, 30). This indicates that TET2 not only maintains endothelial integrity but may also represent a novel therapeutic target for endothelial dysfunction-related vascular diseases. Given the crucial role of endothelial function in the process of thrombosis, we speculate that TET2 may influence thrombus formation by modulating endothelial function.

#### 2.2.3 The role of TET2 in smooth muscle cells

In vascular smooth muscle cells (VSMCs), TET2 has been identified as a key epigenetic regulatory factor for VSMC differentiation and phenotype transition to pro-proliferative and migratory phenotypes (31, 32). TET2 regulates cellular plasticity and differentiation, which are vital for maintaining vascular homeostasis. High levels of TET2 are associated with mature and differentiated SMC phenotypes, while dedifferentiated SMCs show a significant loss of TET2 (33). *in vitro*, experiments have confirmed that overexpression of TET2 increases the level of 5hmC by catalyzing demethylation of 5mC, leading to high expression of the pro-contractile gene MYOCD, maintaining the contractile phenotype, and preventing VSMCs from dedifferentiation and vascular remodeling (Figure 2) (33, 34). In addition, Allison C. Ostriker et al. reported that overexpression of TET2 inhibited IFN *γ*-induced dedifferentiation of VSMCs. TET2 can inhibit apoptosis and abnormal proliferation of VSMCs induced by IFN *γ* and TNF—*α* signalling pathways, thereby preventing intimal thickening (35). These conditions can predispose to vascular complications, including thrombosis (36). Moreover, the knockdown of TET2 in VSMC leads to changes in DNA methylation and results in epigenetic modifications of histones, indicating that TET2 may synergistically regulate DNA accessibility with other chromatin-modifying enzymes (32). Furthermore, TET2 modulates the epigenetic landscape of VSMCs, influencing their response to various stimuli and their ability to adapt to changes in the vascular environment. For instance, studies have shown that TET2 is involved in the differentiation of VSMCs from pluripotent stem cells, underscoring its role in vascular development and repair (37). Thus, the TET2 gene plays an integral role in the proliferation of vascular endothelial cells, ensuring that growth remains within normal limits. This regulatory mechanism is important in maintaining the structural integrity of the vasculature, which is crucial in the prevention of thrombus formation.

#### 2.2.4 The interaction of TET2 with coagulation factors

The TET2 gene serves as a critical regulator in venous thrombosis by modulating the interaction of coagulation factors. Accumulating evidence indicates that TET2 epigenetically regulates the expression of both procoagulant and anticoagulant factors, thereby maintaining the dynamic balance of the coagulation cascade. Experimental studies have demonstrated that loss of TET2 function leads to aberrant platelet activation and enhanced thrombin generation, ultimately increasing thrombotic risk (38). Furthermore, TET2 plays a pivotal role in preserving the coagulation-anticoagulation equilibrium by modulating the expression of coagulation-related genes. Dysfunction of TET2 can induce a hypercoagulable state, which is particularly prominent in patients with myeloproliferative neoplasms (MPNs), where TET2 mutations are frequently observed. Clinical studies have confirmed that these patients exhibit a higher incidence of thrombotic and thromboembolic events, further underscoring the essential role of TET2 in coagulation regulation (39, 40).

#### 2.2.5 TET2 and inflammatory response

It is well established that inflammatory responses play a pivotal role in thrombogenesis. Current evidence suggests that TET2 plays a role in regulating inflammation through epigenetic mechanisms (41, 42). Experimental studies by Fuster et al. demonstrated that TET2 deficiency in macrophages enhances NLRP3 (NOD-like receptor protein 3, LRR-, and pyrin domain-containing protein 3) inflammasome-mediated interleukin-1β (IL-1β) secretion (43). Clinical investigations further revealed elevated plasma levels of the proinflammatory cytokine interleukin-8 (IL-8) in human subjects carrying TET2 mutations (44). These findings collectively suggest that TET2 may function as a negative transcriptional regulator of inflammatory responses. Moreover, somatic TET2 mutations have been shown to promote macrophage inflammatory polarization through clonal hematopoiesis, leading to increased secretion of proinflammatory mediators, including interleukin-6 (IL-6) (43, 45). Epigenetically, TET2 suppresses the transcriptional activation of inflammatory genes, such as IL-6, in dendritic cells and macrophages through histone deacetylation (46). Taken together, we hypothesize that TET2 deficiency may disrupt inflammatory homeostasis, resulting in hyperinflammatory states that ultimately elevate the risk of thrombosis.

#### 2.2.6 TET2 and neutrophil extracellular traps

In experimental models of deep vein thrombosis, neutrophil extracellular traps (NETs) have been firmly established as crucial mediators in the initial stages of thrombus formation (47). By creating a chromatin-based scaffold, NETs provide a structural foundation that enhances platelet adhesion (48). Given their pivotal role, NETs may offer new targets for the development of DVT drugs (49). The architecture of NETs is the primary feature disrupted by TET2 mutations. Neutrophils with TET2 mutations produce smaller and more compact NETs, and the chromatin from these mutated cells exhibits decreased endonuclease accessibility (50). Moreover, NETs produced by TET2-mutated neutrophils are more resistant to degradation by circulating nucleases, suggesting that they may persist longer and drive inflammation. Therefore, TET2 mutations can influence thrombus formation by affecting the structure and function of NETs.

### 2.3 TET2 gene mutations and VTE risk

#### 2.3.1 Epidemiological studies of TET2 mutations

Currently, a growing number of epidemiological studies are being conducted on the association of mutations in the TET2 gene with the risk of VTE. The TET2 gene is implicated in epigenetic regulation-DNA methylation-which is a crucial process in both hematopoiesis and immune function. Recent studies identified TET2 mutations as one of the highly prevalent among various hematological malignancies and clonal hematopoiesis of indeterminate potential (CHIP) (51). These are a series of mutation types, the most common being amino acid substitution, frameshift mutation, nonframeshift deletions, and the creation of premature termination codons. All of these mutations interfere with gene function leading to disease. In a cohort study of older adults, it was found that TET2 mutation was significantly associated with VTE risk (52). The TET2 gene mutations have also emerged as a significant contributor to thrombosis in myeloproliferative neoplasms-a hematologic malignancy (40). Additionally, TET2 gene mutation may independently predict thrombosis in patients with polycythemia vera (53, 54). TET2 gene mutations confer enhanced cardiovascular events; to date, this has been associated with VTE, especially in clonal hematopoiesis amongst elderly subjects (55). Further study has recorded that TET2 mutations can give rise to an inflammatory predisposition for thrombosis (56). This finding identifies a genetic predisposition that may mandate testing of populations with a high risk of developing VTE. Identification of TET2 mutations will enable clinicians to better assess individual risk and implement tailored prevention in a bid to reduce the incidence of VTE.

#### 2.3.2 Impact of mutations on VTE pathogenesis

Mutations in TET2 cause the TET2 enzyme to lose its function, which in turn results in changes to DNA methylation patterns (57). This mutation is expressed in an increased linking of LFA and platelet activation and production of thrombin, leading to an increased incidence of VTE (58). Here, we found that TET2 affects coagulation function. The analysis of a cohort of patients made by Wang Z et al. reported that TET2 mutation patients had significantly elevated D-dimer, significantly decreased AT-III, and increased levels of FDP as compared to normal. This may explain why TET2 mutations are a risk factor for thrombosis in ET patients (38). In addition, TET2 gene mutation was observed to elevate macrophage migration inhibitory factor (MIF) that enhances inflammation and promotes coagulation activation (59). This study raises the possibility that the genetic changes involved in inflammation may be related to the enhanced susceptibility to VTE for patients with mutated TET2 genes. They could also be a part of the process of thrombosis in a number of clinical settings such as those related to cancer (60).

#### 2.3.3 Interaction between TET2 and other genetic factors

Notably, the current study underscores the need for understanding the association of TET2 mutations with other genetic factors in an effort to predict recurrent VTE risk appropriately. Research has indicated the possibility that TET2 mutations are compatible with other mutations for example the FLT3 and JAK2 gene mutations. It is already understood that FLT3 is linked with leukaemia and thrombosis (61), whereas JAK2 mutations act as significant risk drivers for further disease and new VTE events (62). On the other hand, the outcome of TET2 gene mutations on the risk of VTE can be skewed by lifestyle and other environmental factors indicating a reciprocalrogenesis relationship between genotype and exogenous factors (63). Both the JAK2V617F and MPL190A mutations appear to enhance the risk of thrombotic events and the simultaneous presence of both mutations may also enhance the risk; however, the JAK2V619F mutation has not been associated with an increased risk of VTE in clonal haematopoietic individuals, thereby mandating the need to systematically assess VTE risk in clonal haematopoietic patients. The knowledge of these interactions might contribute toward the design of patient patient-tailored therapeutic approach to enhance the outcome in TET2-related RARS patient groups at risk of VTE.

### 2.4 The potential of TET2 as a therapeutic target

#### 2.4.1 Existing therapeutic strategies targeting TET2

TET2 is a key member of the ten-eleven translocation (TET) enzyme family and plays a critical role in DNA demethylation. Its involvement in several hematological malignancies, particularly acute myeloid leukemia (AML), is well documented (64). Current therapeutic strategies for TET2-associated diseases are aimed at restoring its normal function or mitigating factors that reduce its activity. Retinoic acid (RA) and vitamin C activate TET2 transcription, enhancing 5hmC production in immature embryonic stem cells (65, 66). This TET2 activation improves cancer immunotherapy efficacy against renal cell carcinoma, regulates hematopoietic stem cell frequency, and reduces leukaemia occurrence (67, 68). Ascorbic acid and other TET2 modulators play important roles in the treatment of TET2-related diseases, such as inflammatory conditions and malignancies (69, 70). Overall, the development of targeted therapies aimed at TET2 offers hope for improving treatment outcomes in patients with TET2-related malignancies. Therefore, we speculate that targeted TET2 therapy could also be applied to the prevention and treatment of venous thrombosis.

#### 2.4.2 Prospects for novel drug development

The potential for novel drug development targeting TET2 is expanding, particularly in the context of its role in cancer and immune response modulation. Recent research has highlighted the importance of TET2 in regulating the tumour microenvironment and immune evasion mechanisms. For example, TET2 deficiency in immune cells has been shown to exacerbate tumour progression by increasing angiogenesis in lung cancer models (71). This relationship underscores the potential for developing drugs that not only target TET2 directly but also enhance its function within immune cells to improve anti-tumour immunity. Additionally, TET2 has been identified as a key player in the resistance mechanisms of various cancers, such as non-small cell lung cancer, suggesting that drugs aimed at restoring TET2 function could be effective in overcoming therapeutic resistance (70). Drug repositioning strategies, where existing medications are tested for efficacy against TET2-related pathways, may also yield promising results, as evidenced by the ongoing investigations into the effects of glucocorticoids on TET2 activity (69). As research progresses, the development of TET2-targeted therapies could significantly impact cancer treatment paradigms.

#### 2.4.3 The relationship between TET2 and personalized therapy

The relationship between TET2 and personalized therapy is becoming increasingly significant, particularly with the increasing application of genomic profiling of tumours. TET2 mutations and alterations in its expression levels are being recognized as critical factors in determining patient prognosis and treatment response. For instance, in AML, TET2 mutations have been linked to specific therapeutic responses, indicating that patients with these mutations may benefit from tailored treatment approaches (72). Furthermore, the integration of TET2 status into predictive models can enhance the stratification of patients, thereby facilitating individualized treatment plans that consider the unique genetic landscape of each tumour (73). With the evolution of personalized medicine, the role of TET2 as a biomarker for therapy selection is likely to expand, offering new avenues for optimizing treatment efficacy and minimizing adverse effects. The ongoing research into TET2's role in immune modulation and drug resistance further emphasizes its potential as a target for personalized therapeutic strategies, ultimately aiming to improve outcomes for patients with TET2-related malignancies.

### 2.5 Future research directions

#### 2.5.1 Research gaps between TET2 and VTE

TET2, a member of the ten-eleven translocation (TET) family of enzymes, is essential for DNA demethylation and has been linked to various blood cancers. However, research has not established the definite part played by TET2 in the development of VTE. This then may have revealed that mutations in the TET2 gene contribute to blood cell formation problems with an increased risk of thrombosis. However, strong studies which established direct relationships between TET2 abnormality and VTE outcomes cannot be demonstrated. In subsequent studies, more attention should be paid to understanding how TET2 gene mutations can raise the risk of thrombosis: the changes in the regulative or mediatory functions of coagulation and inflammatory processes. Furthermore, the study of TET2 gene mutation frequency in VTE patients, and the ability of mutations to predict thrombosis is necessary. Closing these gaps will enhance risk prediction hence enhancing the opportunity for designing better tailored approaches to the treatment of patients at risk for VTE (74).

#### 2.5.2 Application of multi-omics approaches in TET2 research

The advances in genomics, transcriptomics, proteomics, and metabolomics present a new opportunity to study the biological aspects of TET2 with haematological disorders, especially VTE. These integrated strategies provide the researchers with the opportunity to study the molecular interfaces that are involved in following TET2 mutations and the effects on cellular signalling and metabolism. That is, transcriptomics may provide information about changes in gene expression consequent to TET2 loss-of-function mutations, while proteomics may define alterations in the protein interactions responsible for the prethrombotic condition. Furthermore, MD could reveal metabolomics dysregulation that increases VTE risk. Together with the currently available resources, these datasets allow for the understanding of molecular mechanisms of the effect of TET2 mutations on the risk of thrombosis and open the prospect of the discovery of new targets and biomarkers for VTE (75).

#### 2.5.3 Recommendations for clinical trial design

However, conducting clinical trials for TET2 gene mutations in VTE is full of opportunities as well as challenges. In future trials, more emphasis should be given to genetically structured cohorts in order to evaluate the contribution of TET2 mutations to the risk and outcome of VTE. Multi-centre trials are particularly desirable when large numbers of patients from different settings and the full range of severities of the condition are likely to be enrolled in the trial, so increasing the generalisability of its findings. Finally, the design of biomarker-oriented endpoints, including the evaluation of TET2 mutation and other related molecular markers, will add significant information for predicting treatment efficacy and patient categorization. Moreover, using adaptive trial designs that enable interim changes according to results will enable the exploration of therapeutic prospects for manipulating the pathways impacted by TET2 mutations. Close cooperation between haematologists, geneticists and clinicians will be essential to improving trial parameters in an effort to ensure the answers to the key clinical inquiries regarding TET2 and VTE are appropriately posed by current and future trials (76).

## 3 Conclusion

In conclusion, the important role of TET2 in venous thromboembolism (VTE) marks it as a key focus of haematological and vascular biology research. New information indicates that TET2 mutation and its alterations play a role in the pathogenesis of VTE mainly through epigenetic and inflammatory signaling pathways. This underlines the necessity for more detailed research on the molecular effect of TET2 on thrombosis and its interactions with genetic and environmental factors. The current literature concerning the association of VTE with somatic mutations in the TET2 gene is rather diverse in terms of outcomes and, therefore, not completely coherent. While some papers propose that TET2 has an antithrombotic effect, others point to a prothrombotic effect or a more subtle effect of TET2 on the development of thromboembolism. These different views must then be integrated in order to create the aetiologic narrative of TET2 in VTE that provides a framework for understanding the epidemiologic and clinical realities. Subsequent research should seek to integrate these various sources of information, possibly through very large-scale genomic assessments and extensive mechanisms employed to identify the TET2 function in the vessels. Secondly, there are interesting clinical considerations with regard to TET2 studies. The search for a detailed understanding of the TET2 mechanism of action provides a chance for target therapy. However, this chance faces several specific difficulties such as the absence of deep insights into the time and circumstances of TET2 regulation. Further, understanding the successful rate of interventions focusing on TET2 in preclinical studies and clinical research is essential for applying said findings clinically.

In conclusion, as the current practice of studying the role of TET2 in VTE is enhanced, some questions remain unanswered. Consequently, it is required that further investigations and other subsequent research aim to define the biological significance of TET2 and to define its therapeutic functionality. Understanding TET2 in this manner and reconciling the results from the present study with the findings of other groups, will allow for improved approaches to the primary and secondary prevention of VTE.
